# A Molecular Mechanism for Direct Sirtuin Activation by Resveratrol

**DOI:** 10.1371/journal.pone.0049761

**Published:** 2012-11-21

**Authors:** Melanie Gertz, Giang Thi Tuyet Nguyen, Frank Fischer, Benjamin Suenkel, Christine Schlicker, Benjamin Fränzel, Jana Tomaschewski, Firouzeh Aladini, Christian Becker, Dirk Wolters, Clemens Steegborn

**Affiliations:** 1 Department of Biochemistry, University of Bayreuth, Bayreuth, Germany; 2 Department of Physiological Chemistry, Ruhr-University Bochum, Bochum, Germany; 3 Department of Analytical Chemistry, Ruhr-University Bochum, Bochum, Germany; 4 Institute of Biological Chemistry, University of Vienna, Vienna, Austria; George Washington University, United States of America

## Abstract

Sirtuins are protein deacetylases regulating metabolism, stress responses, and aging processes, and they were suggested to mediate the lifespan extending effect of a low calorie diet. Sirtuin activation by the polyphenol resveratrol can mimic such lifespan extending effects and alleviate metabolic diseases. The mechanism of Sirtuin stimulation is unknown, hindering the development of improved activators. Here we show that resveratrol inhibits human Sirt3 and stimulates Sirt5, in addition to Sirt1, against fluorophore-labeled peptide substrates but also against peptides and proteins lacking the non-physiological fluorophore modification. We further present crystal structures of Sirt3 and Sirt5 in complex with fluorogenic substrate peptide and modulator. The compound acts as a top cover, closing the Sirtuin’s polypeptide binding pocket and influencing details of peptide binding by directly interacting with this substrate. Our results provide a mechanism for the direct activation of Sirtuins by small molecules and suggest that activators have to be tailored to a specific Sirtuin/substrate pair.

## Introduction

Sirtuins are protein deacetylases and hydrolyze one NAD^+^ for each Lys residue they deacetylate [1], which links their activity to cellular energy levels [1],[2]. Sirtuins are involved in regulation of metabolism and stress responses, and they appear to mediate the life-extension and stress resistance effects of caloric restriction (CR), a severe reduction in calorie intake [2],[3],[4]. These roles of Sirtuins have spurred interest in the mechanisms regulating their activity and in small-molecule modulators for therapy [3],[4].

Mammalia have seven Sirtuin isoforms, Sirt1 to Sirt7 [1],[5]. Sirt1, 6, and 7 are mainly nuclear and strong evidence links Sirt1 and Sirt6 to aging processes and stress responses, and Sirt1 also to caloric restriction effects [6],[7],[8]. Three Sirtuins, Sirt3, 4, and 5, are located to mitochondria [9]. Sirt3 was linked to aging processes [10] and regulates central enzymes of energy metabolism [11],[12],[13], indicating how this Sirtuin could also contribute to caloric restriction effects. Sirt5, which is predominantly expressed in lymphoblasts and heart muscle and suggested to contribute to malignant diseases [14], regulates carbamoyl phosphate synthetase 1 (CPS1), a key enzyme of the urea cycle [15],[16]. Sirt5-dependent deacetylation activates CPS1 and is increased through a protein-rich diet, during fasting, and during long term CR, indicating that Sirt5 might contribute to CR effects. Sirt5 has an even higher activity against malonylated and succinylated substrates *in vitro* and desuccinylates a CPS1 site *in vivo* [17], but the physiological function of this deacylation remains to be established.

Sirtuins are attractive drug targets, and the compounds and mechanisms regulating their activity are intensely studied [3],[4]. The conserved Sirtuin catalytic core comprises a Rossmann fold domain and a Zn^2+^-binding domain [18]. The substrate polypeptide occupies a cleft between these domains, with the acetyl-Lys in a tunnel leading to the active site. There, it reacts with NAD^+^ under nicotinamide release to an alkylimidate intermediate, which is finally hydrolyzed [1]. Most pharmacological inhibitors appear to block the NAD^+^ or peptide site and often show moderate potency and isoform specificity [19],[20],[21]. The product nicotinamide, in contrast, acts as a general physiological Sirtuin inhibitor via a non-competitive base-exchange mechanism [1]. Isonicotinamide can relieve this inhibition and thus act as an apparent, non-specific Sirtuin activator [22]. A more isoforms-selective Sirtuin activator is resveratrol, a plant-derived polyphenol that can stimulate yeast Sir2 and mammalian Sirt1 [23] yet also affects other targets. Resveratrol and other Sirtuin activating compounds (STAC; [24]) can induce effects similar to CR [25],[26], including Sirtuin-mediated lifespan extension [23],[27] and protection against diseases such as insulin resistance [28],[29]. Resveratrol affects, however, a variety of proteins and signaling pathways [27],[30],[31],[32] and was shown to thereby also indirectly induce Sirtuin activation. The development of direct Sirtuin activators with improved bioavailability and specificity has been hampered by a missing understanding of the molecular mechanism of Sirtuin activation [4],[33]. In particular, no rationale is available why the stimulating effect is observed with peptides carrying a fluorophore C-terminal to the deacetylation site and only with some, but not all, other substrates [31],[34],[35],[36].

Here, we show that resveratrol can directly activate the deacetylase activity of Sirt5 and inhibit the enzyme’s desuccinylase function as well as Sirt3-dependent deacetylation. The effects are observed for peptides and complete protein substrates and are independent of fluorophore-modifications. We then used Sirt5 and Sirt3 as model systems to investigate the molecular mechanisms of resveratrol-dependent Sirtuin modulation. Crystal structures of ternary Sirt5 and Sirt3 complexes with fluorogenic peptide and compound reveal a direct contact to the substrate fluorophore, which indicates a mechanism for Sirtuin modulation by resveratrol-like compounds and suggests a general substrate-dependence for their effects.

## Results

### Resveratrol Activates Human Sirt5

**Figure 1:**
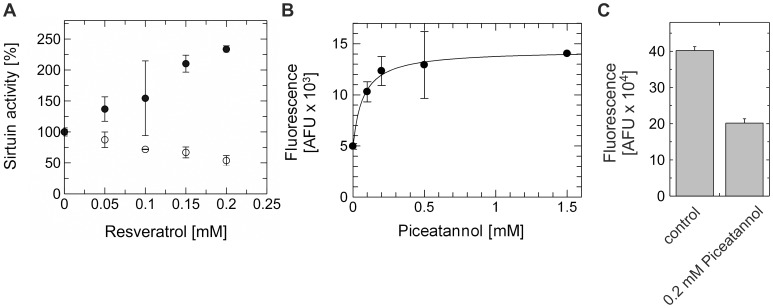
Effects of resveratrol and piceatannol on the FdL-peptide deacetylation activities of Sirt3 and Sirt5. **A** Deacetylation activity of Sirt3 against FdL2 (○) and Sirt5 against FdL1 (•) determined at increasing resveratrol concentrations. Activities are given relative to the value in absence of resveratrol. **B** Dose-response experiment for the piceatannol-dependent stimulation of FdL1 deacetylation by Sirt5. **C** Inhibition of Sirt3 FDL2 deacetylation activity by piceatannol. Error bars represent standard deviations.

Sirt1 is the only mammalian Sirtuin isoform that has been shown to be activated by resveratrol [23], while weak inhibitory effects were mentioned, without details, against Sirt2 and Sirt3 [24],[34]. We tested resveratrol effects on the activities of two mitochondrial Sirtuins, human Sirt3 and Sirt5. We found that 0.2 mM resveratrol can stimulate the Sirt5 deacetylase activity against the fluorophore-modified peptide substrate “Fluor-de-Lys” (FdL) 1 about 2.5-fold (Figure 1A). This effect was isoforms-specific, since 0.2 mM resveratrol inhibited Sirt3-dependent deacetylation of FdL1 (data not shown) and FdL2 (Figure 1A). The dose-response curves show that high µM compound concentrations are necessary for significant effects, similar to Sirt1 activation (EC_50_ ∼ 0.05–0.1 mM, ∼2-8-fold activation depending on the assay used [23],[24]).

Due to the low solubility of resveratrol in water we could not achieve full inhibition of Sirt3 and maximum stimulation of Sirt5. The low bioavailability of resveratrol has prompted speculations that its metabolites might be responsible for the *in vivo* effects [30], and a number of resveratrol-related compounds indeed activate Sirt1 [23]. We tested piceatannol, a resveratrol metabolite that carries an additional hydroxyl group and shows higher solubility (Table S1), and found that it also stimulates Sirt5 (Figure 1B) and inhibits Sirt3 (Figure 1C) activity in the FdL peptide assay. A piceatannol dose-response experiment revealed an EC_50_ of 0.07±0.02 mM for Sirt5 stimulation (Figure 1B). Thus, Sirt5 can be activated by resveratrol and piceatannol, with a potency comparable to that reported for Sirt1, making this system suitable for studying the molecular mechanism of Sirtuin activation.

### Sirt5 Complex With Fdl1 Peptide And Resveratrol

**Figure 2:**
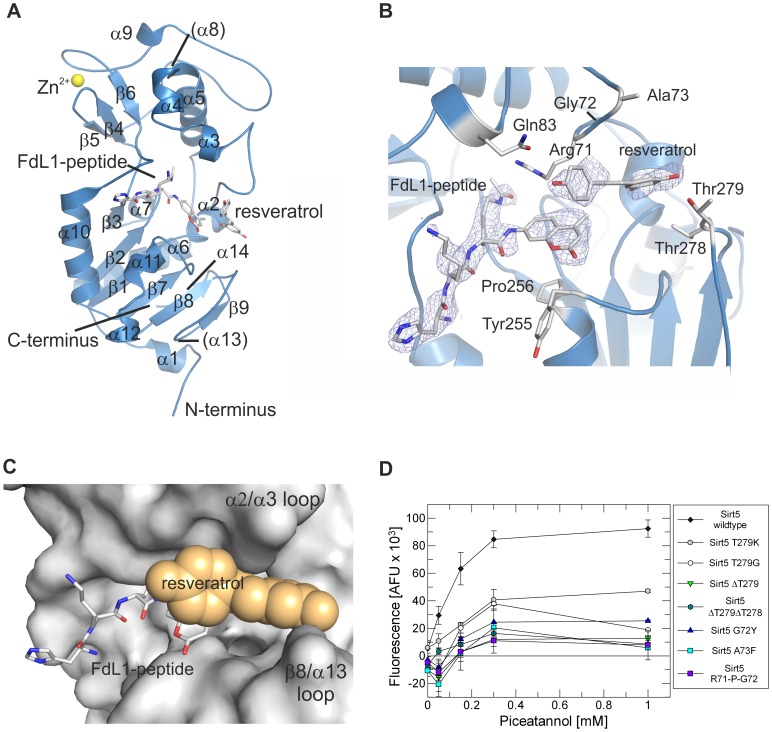
Crystal structure of human Sirt5 in complex with FdL1-peptide and resveratrol. **A** Overall structure of the Sirt5/FdL1/resveratrol complex. Secondary structure elements are labeled as in [42] (elements not present here are in parentheses). **B** Closer view of the activator binding site, showing the direct interaction between substrate peptide and resveratrol. The 2F_O_–F_C_ electron density is contoured at 1σ. **C** Surface representation of the Sirt5 ligand binding sites, showing that the activator closes the peptide channel entrance. The peptide is shown as sticks, the activator as calotte model in orange. **D** Piceatannol dose response experiments showing the changes in stimulated Sirt5 FdL1 deacetylation activity upon mutation of residues in the resveratrol binding loops. The assignment of symbols to Sirt5 residue replacement mutations, deletions (Δ), or a single residue insertion (R71-P-G72: Pro insertion) is indicated on the right side.

To rationalize the mechanism of Sirt5 activation in the FdL assay, we solved a crystal structure of Sirt5 in complex with FdL1 substrate peptide and resveratrol (Table 1). Data quality was impaired by significant twinning (twin fraction 47%; Figure S1), and the density for resveratrol further weakened by its low occupancy (∼50%), likely due to its low solubility. However, density not accounted for by the built Sirt5/FdL1 model indicated an activator molecule bound close to the peptide substrate (Figure 2A,B), directly contacting the FdL fluorophore. To confirm the significance of the weak ligand density despite of the low overall quality, we positioned ligand molecules at other sites harboring noise or solvent density. No significant 2Fo–Fc densities but strong negative Fo–Fc densities at these positions after refinement supported the resveratrol site in our structure (Figure S2). This position in direct contact with the peptide fluorophore is further supported by the structure of a Sirt3/FdL1-peptide/piceatannol complex (see below).

**Table 1:** Diffraction data collection and refinement statistics.

	Sirt5/FdL1/resveratrol	Sirt3/FdL1/piceatannol
Space group	P2_1_	R32
Unit cell constants	a = 41.3 Å, b = 112.7 Å c = 55.9 Å; β = 90.1°	a = b = 114.6 Å, c = 123.7 Å
Resolution (Å)	38.8–2.6	38.7–2.3
Unique reflections	15354	14069
<I/σ>(outermost shell)	14.5 (3.0)	20.0 (4.3)
Completeness (outermost shell)	97.6 (93.4)	99.9 (99.9)
R_merge_ (a) (outermost shell) (%) R_meas_ (b) (outermost shell) (%)	7.5 (45.4) 8.8 (53.6)	8.4 (48.7) 9.1 (52.7)
Resolution range for refinement (Å)	38.8–2.6	38.7–2.3
Total reflections used	15353	13365
Number of atoms in asymmetric unit		
Protein	4111	2068
Ligands	63	78
Water	15	103
R.m.s. deviations:		
Bond length (Å)	0.01	0.02
Bond angles (°)	1.3	1.9
Average B factor (Å^2^)		
Protein	40.6	31.3
Peptide	62.2	34.5
Resveratrol/Piceatannol	63.2	35.2
Zinc ions	32.2	21.8
Final R_cryst_/R_free_^(c)(d)(e)^ (%)	19.9/25.2 ^(twinned R values)^	17.4/24.1

(a) *I* is the intensity of an individual measurement and the corresponding mean value.

(b) for details see [62]_._

(c) is the observed and the calculated structure factor amplitude.

(d) R_free_ was calculated from 5% of measured reflections omitted from refinement and not related by the twinning law to reflections of the R_cryst_ set.

doi:10.1371/journal.pone.0049761.t001

In the Sirt5/FdL1-peptide/resveratrol structure, the asymmetric unit contains two Sirt5 monomers with the active centers facing each other. Due to limited space resulting from this orientation only one peptide binding cleft is occupied by FdL1-peptide. The acetyl-Lys points toward the catalytic His158 [37], and the FdL1 N-terminus occupies the same peptide binding channel as observed in other Sirtuin/peptide complexes [1],[38] (Figure 2B). The C-terminal FdL1 fluorophore is packed with one surface on top of a hydrophobic patch formed by Tyr255 and Pro256 in the β7/α11 loop, and its edge is approached by Arg71. Its second plane would be largely solvent accessible in the absence of the activator. However, resveratrol is positioned next to the peptide and directly contacts this fluorophore plane (Figure 2B,C). The activator A-ring is bound between Thr278 and Thr279 in the β8/α13 loop on one side, and Gly72 and Ala73 in the α2/α3 loop on the other side. The resveratrol B-ring interacts in almost perpendicular orientation with the coumarin fluorophore and contacts a protein patch formed by Gln83 and the α2/α3 loop around Arg71 and Gly72. Consistent with the resveratrol interaction with the β8/α13 and α2/α3 loops, mutations in these Sirt5 regions reduced stimulation. Replacing Thr279 with a bulkier Lys restricted stimulation to about 50% of stimulated wildtype activity, and removing the side chain (Thr279Gly) to about 20–30% (Figure 2D). Deleting the entire residue alone or in combination with Thr278 limited stimulation to ∼10%. Likewise, replacing Gly72 by a bulkier Tyr or Ala73 by a Phe yielded stimulation of ∼25% and 10–20% of wildtype stimulation, respectively. Extending this loop by inserting a proline (Arg71-Pro-Gly72) reduced stimulation to about 10% of wildtype activation.

The structure indicates that binding of resveratrol between the α2/α3 and β8/α13 loops closes the active site opening (Figure 2C), thereby trapping the bound peptide and increasing the interaction interface by directly contacting the FdL1 substrate fluorophore. This interaction apparently leads to a substrate binding mode more suitable for the subsequent reaction step (see below).

### Sirt3 Complex With Peptide And Piceatannol

**Figure 3:**
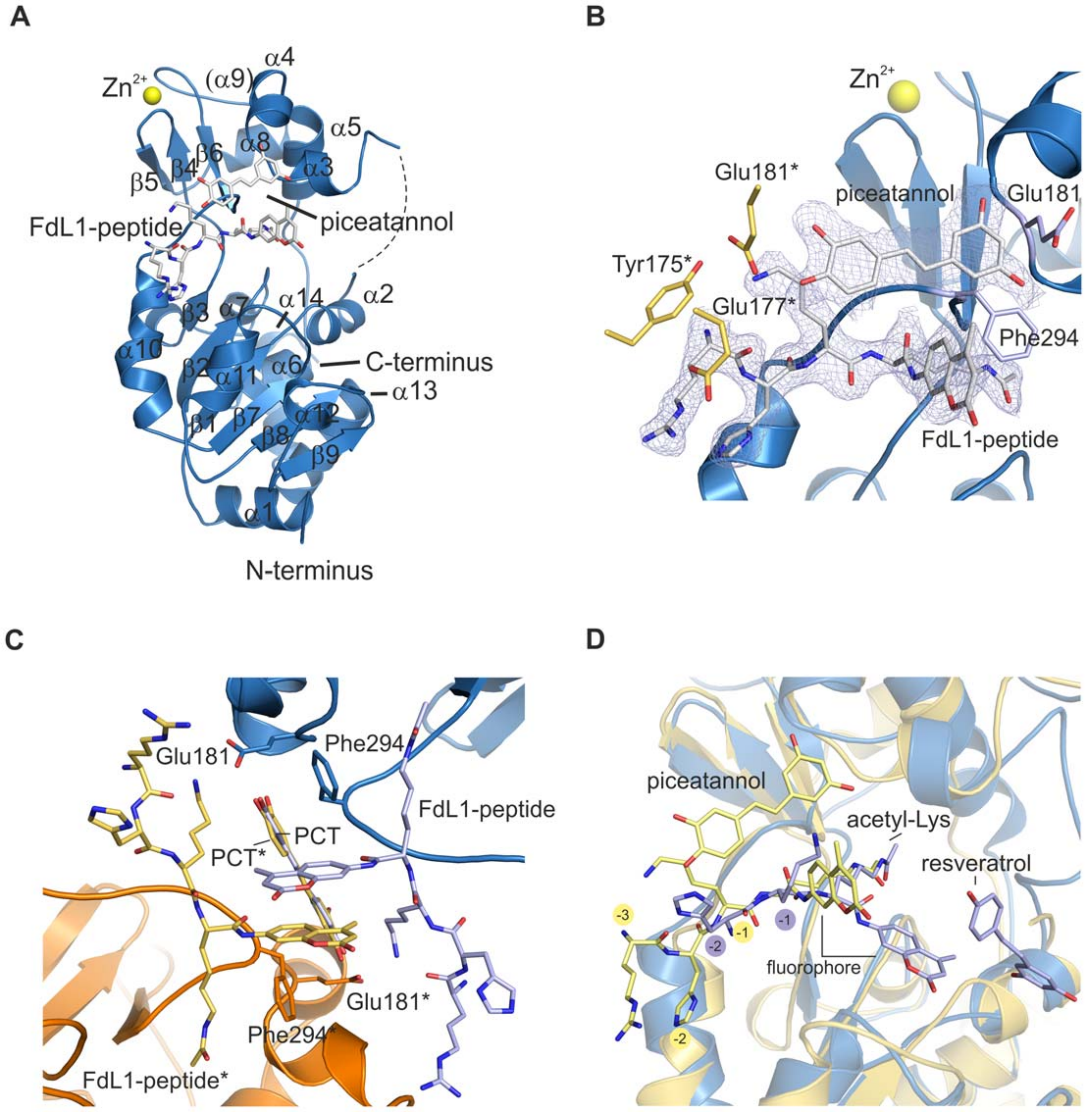
Crystal structure of human Sirt3 in complex with FdL1-peptide and piceatannol. **A** Overall structure of the Sirt3/FdL1/piceatannol complex. Secondary structure elements are labeled according to the Sirt5/FdL1/resveratrol complex for comparison. **B** Closer view of the activator binding site, showing the direct interaction between substrate peptide and piceatannol similar to the arrangement in the Sirt5/FdL1/resveratrol complex structure. The 2F_O_-F_C_ electron density is contoured at 1σ. Residues of the symmetry related Sirt3 monomer (*) contributing to piceatannol binding are shown in yellow. **C** Interface between Sirt3 (blue) and its symmetry related monomer (orange) showing the binding mode for the FdL1-peptide and piceatannol. Ligands and residues involved in their binding are presented as blue and yellow sticks. **D** Superimposition of Sirt5/FdL1/resveratrol (blue) and Sirt3/FdL1/piceatannol (yellow). The residues N-terminal to the acetylated lysine are labeled for comparison.

To investigate why resveratrol-like compounds activate Sirt1 and Sirt5 but inhibit Sirt3 we also solved the crystal structure of Sirt3 in complex with FdL1-peptide and piceatannol (Table 1). The asymmetric unit of the Sirt3/inhibitor complex contains one monomer (Figure 3A). As in the Sirt5/activator structure, FdL1-peptide and piceatannol bind in close proximity and directly contact each other (Figure 3A,B,C). Peptide and piceatannol form crystal contacts to a symmetry-related Sirt3 monomer, resulting in π stacking interactions of the coumarin fluorophores of two FdL1-peptides and an interaction of the modulator with both stacked aromates (Figure 3C). Piceatannol binds perpendicular to the coumarin rings, similar to resveratrol in the Sirt5/activator complex. The hydrophobic binding unit is completed by Phe294 and its symmetry-related counterpart Phe294*. Additionally, Glu181/181* participate in positioning of piceatannol by forming hydrogen bonds to hydroxyl groups in both rings of piceatannol.

The Sirt3/inhibitor complex clearly shows the direct interaction between compound and coumarin moiety of the FdL1-peptide indicated by the Sirt5/activator structure. An overlay of the two complexes (Figure 3D) shows that both acetylated FdL1 lysines are bound in the hydrophobic active site tunnel, but the neighboring fluorophore/modulator pairs are differently arranged. As a consequence, the entire FdL1-peptide is shifted in the Sirt5/activator structure relative to the Sirt3/inhibitor complex, positioning the acetamide group deeper in the hydrophobic binding tunnel. It is tempting to speculate that the interaction of resveratrol/piceatannol with the coumarin moiety is responsible for these differences, resulting in a productive substrate conformation in Sirt5 but in non-productive substrate binding in Sirt3. Although the inhibitor contact to a symmetry-related substrate molecule in Sirt3 is likely a crystallization artifact, the Sirt3 structure shows again that the resveratrol-like compound can bind on top of the substrate and directly interact with the C-terminal peptide fluorophore, apparently influencing the substrate binding mode and presumably thereby inhibiting the subsequent reaction step.

### Structure-Activity Relationship For Activators

The Sirt5/resveratrol and Sirt3/piceatannol structures allow to rationalize structure-activity relationships for Sirtuin modulators. In both complexes, the resveratrol/piceatannol ring A positions 3–5 are solvent exposed (Figure 2B,3B) and allow modifications without steric clashes, consistent with the stimulatory activity of the resveratrol derivative deoxyrhapontin (3,5-dihydroxy-4′-methoxystilbene 3-O-β-D-glucoside) against Sirt1 [23]. The B-ring of the compounds appears crucial for mediating the interaction with the enzyme-bound substrate. We indeed find that tyrosol (4-(2-hydroxyethyl)phenol), a cardioprotective compound from white wine [39] whose scaffold is reduced to the resveratrol B ring and part of the linker (Table S1), activates Sirt5 in a Cytochrome c deacetylation assay (see below, Figure S3A). The activator/fluorophore interaction further explains why changing the resveratrol 4′-position to a thiomethyl group or other small and hydrophobic moieties increases the ability to stimulate FdL1 deacetylation by Sirt1 [36]. Adding instead a bulky and polar acetyl group would lead to unfavorable interactions and abolishes most of the Sirt1 stimulating potency [36]. Even the Sirt1 activators quercetin and fisetin [23], despite of different scaffolds (Table S1), can be aligned with piceatannol to simultaneously superpose the 3′ and 4′ hydroxyl groups and the distal ring system. However, only fisetin shows a weak inhibitory effect on Sirt5 and significant inhibition of Sirt3 in an ELISA-based deacetylation assay (see below), whereas quercetin has no significant effect on Sirt5 and Sirt3 activity (Figure S3B). Thus, despite of similarities between Sirtuins enabling them to share some ligands, such as the activator resveratrol for Sirt1 and Sirt5, details of binding sites and ligand interactions appear sufficiently different to allow for isoform-specific effects, such as Sirt1 activation and differential inhibition of Sirt3 and Sirt5 by fisetin.

### Resveratrol Stimulates Deacetylation Of Non-Modified Peptides And Protein Substrates

**Figure 4:**
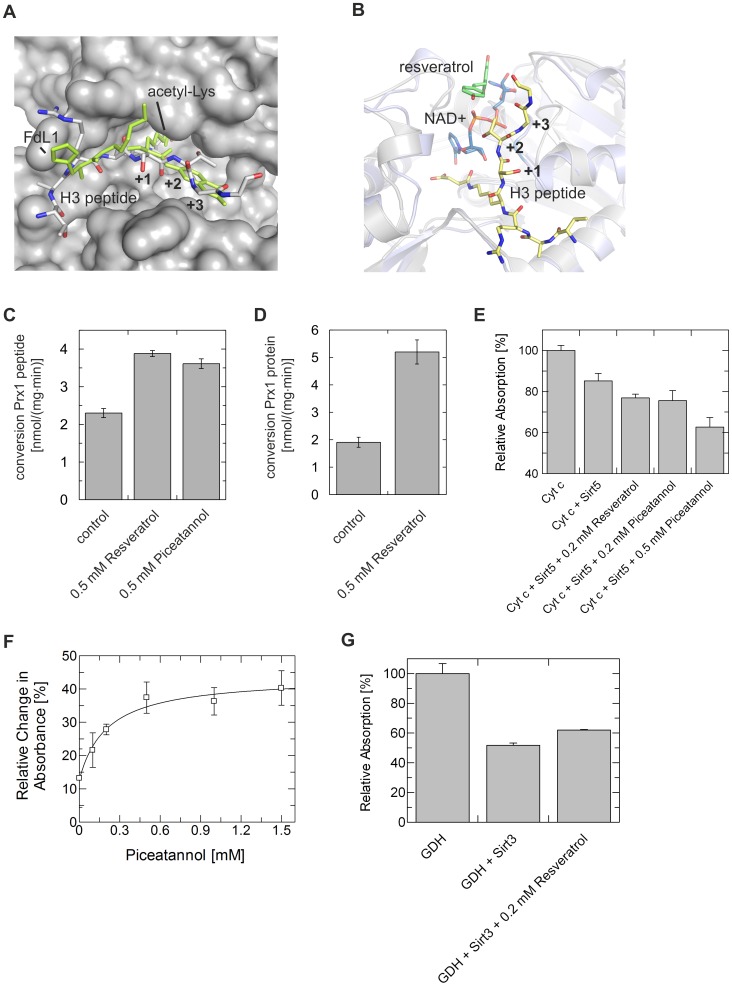
Effects of resveratrol and piceatannol on Sirt3 and Sirt5 deacetylation activities against fluorophore-free peptides and proteins. **A** Overlay of the Sirt5/FdL1/resveratrol (FdL1 in green, activator omitted for clarity) and Sirt5/succinylated H3 peptide/NAD^+^ (H3 peptide in atom type coloring, NAD^+^ omitted for clarity) complexes. The fluorophore occupies the site normally accommodating residues of the substrate polypeptide. **B** Overlay of Sirt5/FdL1/resveratrol (FdL1 omitted for clarity) and Sirt5/succinylated H3-peptide/NAD^+^ complex. Ligands are colored according to atom types with carbon atoms in green (resveratrol), yellow (H3 peptide), and blue (NAD^+^). **C+D** Sirt5-dependent deacetylation of Prx1-Lys197 peptide (**C**) and Prx1 protein specifically acetylated at Lys197 (**D**) is activated by resveratrol-related compounds. **E** Sirt5-dependent deacetylation of Cytochrome c determined in an ELISA shows that resveratrol and piceatannol stimulate this activity (which leads to a loss of signal in this assay). **F** Dose-reponse experiment for the piceatannol-dependent stimulation of Cytochrome c deacetyalation by Sirt5. Shown is the loss in absorption at different piceatannol concentrations relative to untreated Cytochrome c. **G** Sirt3-dependent GDH deacetylation tested in an ELISA (deacetylation decreases absorbance) shows an inhibitory effect of resveratrol. Error bars represent standard errors of linear fits to time-series experiments (C+D) or standard deviations (E–G), respectively.

Comparison of the Sirt5/FdL1/resveratrol complex to Sirtuin complexes with longer substrate peptides, and in particular to a Sirt5/H3 peptide/NAD^+^ complex [17], illustrates that the FdL1 fluorophore accommodating pocket would normally harbor residues of the substrate polypeptide (Figure 4A). In particular, the C-terminal H3 peptide residues +1 (relative to the acylated Lys) to +3 bind to the fluorophore-accomodating Sirt5 patch around Tyr255. The observed activator position closes the entrance of the peptide binding cleft without blocking the peptide binding channel itself or the NAD^+^ binding site (Figure 4B), and it allows resveratrol to contact substrates C-terminal to the acetyl-Lys. It thus reveals how the substrate C-terminus can influence the ability to stimulate deacetylation, explaining why resveratrol-dependent stimulation of Sirt1 activity against FdL peptides depends on the presence of the C-terminal FdL fluorophore [34],[35]: The lost stimulation with non-modified short peptides lacking any C-terminal extension is due to the lost direct contact between activator and substrate. Consistently, activation of Sirt1-dependent deacetylation of peptides containing a polypeptide extension instead of a fluorophore C-terminal to the acetyl-Lys was also reported [24],[36]. To test whether stimulation of Sirt5 activity also applies to completely non-modified peptides and even protein substrates, we first analyzed Sirt5-dependent deacetylation of a non-modified peptide with amino acids C-terminal from the acetyl-Lys by using mass spectrometry. Sirt5 deacetylates a Peroxiredoxin 1 acetylation site (Prx1-Lys197 SKEYFS(acylK)QK) as *in vitro* and putative *in vivo* substrate (unpublished data), and resveratrol as well as piceatannol stimulated Sirt5 activity against Prx1-Lys197 about 1.7-fold (Figure 4C). We next tested for a direct effect of resveratrol-like compounds on Sirtuin-dependent deacetylation of a full-length protein substrate. Full-length Prx1 protein specifically acetylated at Lys197 through native chemical ligation can be deacetylated by Sirt5 (unpublished data), like the Prx1-Lys197 peptide, and addition of resveratrol increased deacetylation of the Prx1 protein about 2.7 fold (Figure 4D). To confirm the stimulatory effect on deacetylation of a substrate protein, we also analyzed the Sirt5-dependent deacetylation of the *in vitro* substrate Cytochrome c [12]. Since the Sirt5-targeted acetylation site in Cytochrome c is unknown, we applied an enzyme-linked immunosorbent assay (ELISA) to analyze the overall acetylation level of this substrate protein [12]. Adding 0.2 mM resveratrol or piceatannol, respectively, increased Sirt5-dependent deacetylation of Cytochrome c (Figure 4E). A dose-response experiment with piceatannol resulted in a ∼3-fold stimulation with an EC_50_ of 0.21±0.08 mM (Figure 4F). In contrast, resveratrol caused weak inhibition when tested on Sirt3 in an analogous ELISA for glutamate dehydrogenase (GDH) deacetylation (Figure 4G), again consistent with the FdL results. These results show that the fluorophore label is not essential for the stimulatory effect of resveratrol on Sirtuin-dependent deacetylation but can be replaced by a regular polypeptide chain, and consistently, that activation can also be observed with a complete protein as a substrate.

### Resveratrol Effects On Sirt5 Depend On The Substrate Sequence And The Type Of Acyl Modification

**Figure 5:**
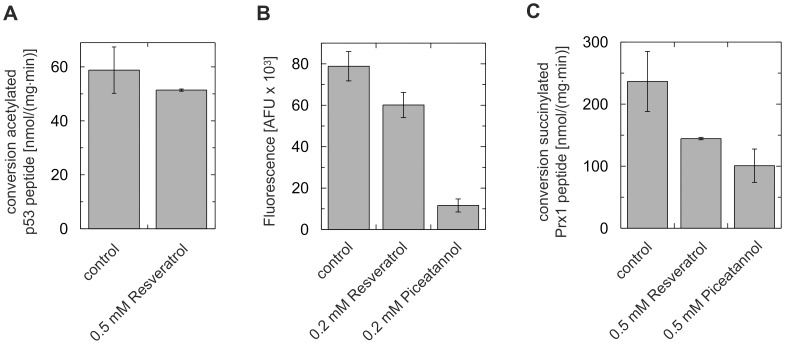
Dependency of the resveratrol/piceatannol effects on the substrate sequence and the type of acyl modification. **A** Sirt5-dependent deacetylation of p53-Lys382 peptide is not affected by resveratrol. **B+C** In contrast to its stimulated deacetylation activity, Sirt5’s deacylation activity against succinylated FdL1 (**B**) and Prx1-Lys197 (**C**) peptides is inhibited by resveratrol and piceatannol. Error bars represent standard errors of linear fits to time-series experiments.

Due to the direct substrate/activator contact revealed by our complex structures, the effects of resveratrol-like compounds should strongly depend on the substrate, consistent with results from Sirt1 deacetylation assays [27],[35]. In particular, the effect on extended polypeptides should depend on the substrate sequence due to the direct deduced contacts to residues in the +1 to +3 positions. Consistent with this hypothesis, resveratrol activates Sirt5-dependent deacetylation of FdL1-peptide, Prx1-Lys197, and Cytochrome c, but has no effect on deacetylation of p53-Lys382 (RHK(acylK)LMFK; Figure 5A). This peptide corresponds to FdL1 (RHK(acylK)-fluorophore) in the N-terminal sequences but carries amino acids instead of a fluorophore on the C-terminal side, highlighting the importance of the C-terminal extension for the compound effect. Consistently, changing the C-terminal fluorophore to different single amino acid residues resulted in different STAC effects - stimulation, inhibition, or no effect - on Sirt1-dependent deacetylation [41].

We recently found that the Sirt5 deacylation activities deacetylation and desuccinylation are differently regulated by nicotinamide [40]. Since details of the Sirtuin/resveratrol interaction are influenced by the substrate, we speculated that the type of acyl group at the lysine should also influence the effect of resveratrol on Sirt5. Indeed, we find that Sirt5-dependent deacylation of succinylated FdL1 and Prx1-Lys197 peptide is inhibited, in contrast to the stimulation observed with the acetylated forms of these peptides (Figure 5B,C). Since Sirt5 binds succinylated substrates in a more defined orientation through an additional salt bridge [17], we assume that resveratrol improves a low-productive binding mode of acetylated substrates but disturbs the already productive binding orientation of succinylated ones. Thus, in general, Sirtuin modulation by polyphenols appears to strongly depend on a compatible substrate/activator pair.

### A Model For Sirtuin Regulation By Resveratrol-Like Compounds

**Figure 6:**
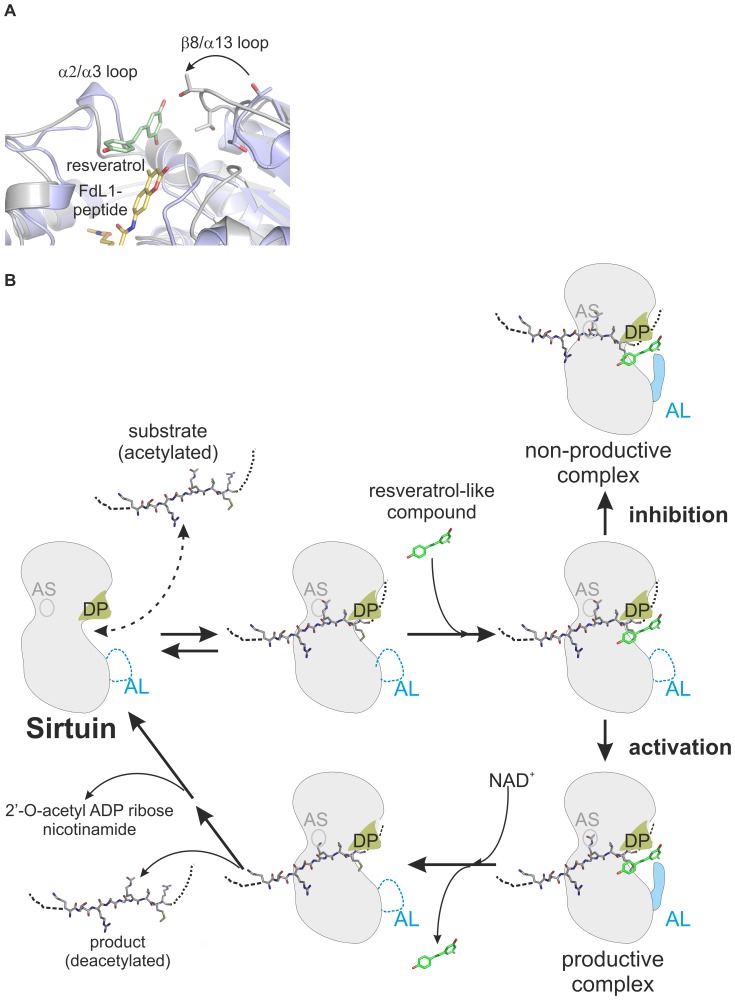
Model for Sirtuin regulation by resveratrol-like compounds. **A** Superimposition of Sirt5/FdL1/resveratrol and Sirt5/succinylated H3 peptide/NAD^+^. The movement of the β8/α13 loop is indicated by an arrow. The succinylated H3 peptide and the NAD^+^ molecule are omitted for clarity. **B** Model for the regulation of Sirtuins by resveratrol-like compounds. After binding of the substrate polypeptide the small molecule attaches to a “docking patch” (DP). It induces ordering of an “adaptable loop” (AL), leading to closure of the peptide exit and stabilization of the enzyme/substrate complex. Depending on the fit between substrate and small-molecule, the substrate is properly oriented in the active site (AS; e.g. Sirt5/Cytochrome c/resveratrol) or adopts a non-productive conformation (e.g. Sirt3/GDH/resveratrol), leading to stimulation or inhibition of turnover, respectively. After deacetylation, the activator dissociates, opening the peptide exit for product release.

Resveratrol/piceatannol binds on top of the Sirtuin-bound substrate peptide. In activator-free Sirt5 (PDB ID 2NYR/2B4Y [42] and 3RIG/3RIY [17]) and other Sirtuin structures in presence or absence of a peptide (Sirt3∶3GLS/3GLR [43]; Sirt2∶1J8F [44]; Sir2Tm: 2H2I/2H2F [38]), the β8/α13 loop is either disordered or in an “open” conformation, leaving a larger gap to the α2/α3 loop. In the Sirt5/FdL1/activator complex, the β8/α13 loop is “closed” and forms part of the activator binding pocket, indicating that resveratrol binding leads to restructuring or stabilization of this loop (Figure 6A). The opposite part of the activator binding pocket – the so-called flexible cofactor binding loop α2/α3– is disordered in absence of the cosubstrate NAD^+^ and adopts an open or closed conformation depending on the ligand species (cosubstrate, intermediate, or product) [45]. For Sirt5 in complex with NAD^+^ or suramin, respectively, the α2/α3 loop is ordered and shows no significant changes upon resveratrol binding. The activator thus appears to contact a prebuilt Sirt5 “docking patch” (formed by α2/α3 loop and substrate) to induce formation of the residual interaction site through stabilization of an “adaptable loop” (β8/α13 loop). Comparison of Sirt3 structures [43] indicates that peptide binding leads to a narrowing of the cleft between the α2/α3 and β8/α13 loops. Peptide binding, besides directly supplying interaction surface for the activator, might thus indirectly contribute to formation of the activator binding site by influencing these regions. The other way round, the loops might mediate effects of the activator on substrate binding and turnover. Consistent with a catalytic and activation-mediating role of the closure movement, Sirtuin structures representing subsequent reaction steps, NAD^+^ binding and intermediate formation (3RIY [17], 3GLT [43], 2QQF [46]), show a further narrowing due to a shift of the α2/α3 loop toward β8/α13. This shift appears to disturb the modulator binding site in Sirt5 and is a possible mechanism for triggering activator release.

To summarize, our model for Sirt5 regulation by resveratrol-like compounds (Figure 6B) involves small molecule binding to a “docking patch” (“DP” in Figure 6B) and an “adaptable loop” (“AL”) of the enzyme/polypeptide complex. These loops and the direct interactions between compound and substrate determine whether a productive or a non-productive substrate state is stabilized, i.e. whether the compound activates or inhibits deacylation. In case of a productive interaction, loop movements during the subsequent reaction steps destroy the modulator binding site, triggering activator release and product dissociation. This mechanism for modulation of Sirt5-dependent FdL1 deacylation also provides a framework for other isoforms and substrates, since it can explain available Sirt1 data and the modulation of Sirt5-dependent deacylation of fluorophore-free substrates described in the present work.

## Discussion

The prominent role of Sirtuins in aging and metabolic regulation renders them attractive drug targets [4]. The Sirt1 activator resveratrol extends lifespan [23],[27] and protects against diseases such as insulin resistance [28],[29]. A link of resveratrol effects to Sirtuins, besides other cellular targets [30],[32], is well documented [23],[26]. Two types of effects, direct Sirtuin activation and indirect effects on Sirtuin regulating pathways seem to contribute [30],[32]. Our data suggest that direct activation and inhibition of Sirt5 and Sirt3 activities, besides Sirt1 activation, might also contribute to physiological effects in mammals, but that remains to be tested. The effects on Sirt3 and Sirt5 also show that resveratrol-like compounds can interact with the conserved Sirtuin catalytic core, instead of the suggested binding to the Sirt1-specific N-terminus [24], consistent with the finding that piceatannol is a potent Sirt2 inhibitor [47]. The low potency of resveratrol against Sirt5 (this study) and Sirt1 [23],[24] is in fact consistent with the high compound concentrations needed to induce many of its *in vivo* effects [27]. Sirtuin activation thus appears an attractive therapeutic approach [3],[4], but more potent, specific, and bioavailable compounds are needed. Our activation model provides a tool for compound development, although it remains to be shown whether the same mechanism applies to the compound effects observed with other Sirtuins and with fluorophore-free substrates. However, our model rationalizes data for several Sirtuin isoforms and the direct Sirtuin activator/substrate contact could resolve a heated debate on the possibility of Sirtuin activation [33]. It explains the requirement for the C-terminal fluorophore for Sirtuin activation against FdL1 substrate [34],[35] and how other C-terminal extensions, in particular regular amino acids, can also enable activation. Consistently, we and others find resveratrol activation also for longer peptides without fluorophore and for entire substrate proteins (this study and [27],[36]). This mechanism also explains how the compound can activate, not affect, or inhibit a Sirtuin depending on the substrate, consistent with assay data (this study and [27],[35],[41]) and the fact that resveratrol shows Sir2-dependent effects in *C. elegans* overlapping with but not identical to effects of Sir2 overexpression [48]. This sequence dependence suggests that for analyzing effects of this compound class on Sirtuin-dependent deacetylation we have to consider each Sirtuin/substrate pair individually, which on the other hand suggests that drugs can be developed that modulate only deacetylation of few specific Sirtuin substrates. The described resveratrol mechanism is in fact related to an alternative model, which suggested that activator and substrate form a complex in solution that then acts as an improved substrate [31]. However, a lack in correlation between substrate/compound interaction and activation [41] and the substantial contribution of the protein to binding surface and outcome (Sirt5 activation versus Sirt3 inhibition) suggest that the modulator/substrate contact is preferentially formed in the enzyme/substrate complex.

In summary, our results reveal a mechanism enabling resveratrol-like compounds to directly activate Sirtuins. They indicate that several mammalian Sirtuin isoforms can be activated and reveal the exciting possibility to develop regulators targeting only specific Sirtuin/substrate pairs.

## Methods

### Chemicals

All chemicals were obtained from Sigma (Saint Louis, USA) if not stated differently. Acetylated peptides and succinylated Prx1-Lys197 peptide were from GL Biochem (Shanghai, PRC).

### Cloning, Recombinant Expression, And Purification Of Sirt3 And Sirt5

The catalytic cores of human Sirt3 (residues 118–399) and Sirt5 (residues 34–302) were PCR amplified and cloned into pVFT3S (Sirt3; Korean patent 10-0690230) and pET151/D-TOPO (Sirt5; Invitrogen, Carlsbad, USA), respectively, resulting in a construct with N-terminal His-tag (Sirt5) or His+thioredoxin tag (Sirt3) and a TEV protease cleavage site. Proteins were expressed in *E. coli* Rosetta2(DE3) cells (Merck, Darmstadt, Germany) in LB medium by adding 0.5 mM isopropyl-β-D-thiogalactopyranosid at OD_600_ 0.8. Cells were incubated over night at 15°C (Sirt3) or 20°C (Sirt5), respectively, disrupted with a Micorofluidizer, cell debris removed by centrifugation, and the supernatant supplemented with 10 mM imidazole and incubated with talon resin for 1 h at 4°C. Resin was washed with 10 volumes 50 mM Tris/HCl, pH 7.8, 500 mM NaCl and 10 volumes 50 mM Tris/HCl, pH 7.8, 200 mM NaCl, 5 mM imidazol. Recombinant proteins were eluted with 50 mM Tris/HCl, pH 7.8, 200 mM NaCl, 250 mM imidazol, subjected to gel filtration on a Superose 12 column (GE Healthcare, Waukesha, USA) in 20 mM Tris/HCl, pH 7.8, 150 mM NaCl, concentrated, and stored at −80°C. Proteins for crystallization were digested with TEV protease over night and tags removed by affinity chromatography on talon resin prior to gel filtration. Sirt3 protein for activity assays was produced with just a His tag as described previously [12].

### Preparation Of Specifically Acetylated Prx1

A Prx1 fragment (residues 1–195) was expressed as a *Mxe* intein/chitin binding domain (CBD) fusion, fused to a synthetic, specifically acetylated peptide representing the Prx1 C-terminus(C(acetylK)QK, Ser196 substitued with Cys), and purified and analyzed by mass spectrometry as described (Rauh et al., unpublished data).

### Peptide Deacylation Assays

Deacylase activity of Sirtuins was tested with commercial fluorescence assay kits (Biomol, Plymouth Meeting, USA) containing the p53-derived substrate peptides RHKacetyl/succinylK (acetylated or succinylated FdL1, respectively, used as Sirt3 and Sirt5 substrates, respectively) and QPKacetylK (FdL2; Sirt3 substrate) with a C-terminally attached fluorophore. Reactions were incubated at 37°C with 0.5 µg Sirt3, 2.0 µg Sirt5 (acetylated FdL1) or 0.2 µg Sirt5 (succinylated FdL1), respectively, 0.1 mM fluorogenic peptide, and 0.5 mM NAD^+^ if not stated otherwise, and reaction product detected according to the instructions of the manufacturer. In control reactions and blanks, the same amount of compound analyzed or solvent used for its stock solution, respectively, was included. All assays were done in triplicates, and results shown are representatives of at least three independent replications.

For the mass spectrometry assay, 0.5 mM peptide was deacetylated by adding 10 µM Sirt5 and 2.5 mM NAD^+^ in presence or absence of 0.5 mM resveratrol or piceatannol, respectively, and incubation at 37°C. Control and compound sample reactions contained the same amount of solvent (2% (v/v) DMSO). After different time points aliquots of the reaction were stopped with 0.25% (v/v) trifluoroacetic acid and analyzed by LC-ESI-MS as described in [40]. Turnover rates and their errors were deduced by linear fitting within initial product formation ranges.

### Protein Deacetylation Assays

Deacetylation of Prx1 protein specifically acetylated at Lys197 was analyzed in 20 mM Tris/HCl pH 7.8, 150 mM NaCl, 2.5 mM NAD^+^, 2% (v/v) DMSO, with 68.8 µM Prx1 and 13.6 µM Sirt5 in the presence and absence of 0.5 mM resveratrol at 37°C. Aliquots of the reaction mixture were stopped after different time points by adding 5 mM nicotinamide, and proteins digested with trypsin in 25 mM NH_4_HCO_3_ pH 8.5, 5 mM nicotinamide for 4 h at 37°C (trypsin to protein ratio 1∶ 20). Peptides were further treated and analyzed as described [40] for quantification of acetylated and deacetylated Prx1 peptides containing Lys197 using the following transitions: SKEYFC**acK**QK 630,30@cid35.00 [751.38–753.38, 914.43–916.43, 1043.48–1045.48], SKEYFC**K**QK 609.30@cid35.00 [709.37–711.37, 872.43–874.43, 1001.47–1003.47], SKEYFC**K** 481.20@cid35.00 [616.28–618.28, 745.32–747.32, 873.41–875.41]. Turnover rates and their errors were deduced by linear fitting initial product formation ranges. Deacetylation of bovine Cytochrome c (by Sirt5) and GDH type I (by Sirt3) purified from mitochondria (Sigma) was tested in an ELISA as described [12]. Blanks and control reactions were done as described for the peptide assays. All assays were done in triplicates, and results shown are representatives of at least three independent replications.

### Crystallization And Structure Solution

Sirt3 (11 mg/ml in 20 mM Tris/HCl, pH 7.8, 150 mM NaCl) and Sirt5 (12 mg/ml in 20 mM Tris/HCl, pH 7.8, 150 mM NaCl, 2 mM DTT) were crystallized in complex with FdL1-peptide and modulator after pre-incubation with FdL1 (Sirt3, 3 mM; Sirt5, 4 mM) and resveratrol (Sirt5, 1 mM in 5% (v/v) EtOH) or piceatannol (Sirt3, 1 mM in 10% (v/v) DMSO) for 1 h on ice. Protein solution was mixed with reservoir (Sirt3, 200 mM NaCl, 100 mM HEPES pH 7, 10% (v/v) isopropanol; Sirt5, 150 mM NH_4_Cl, 15% (w/v) PEG 3350) supplemented with the same amounts of peptide and activator in a 1∶1 ratio and equilibrated against 0.4 ml reservoir at 20°C. Sirt3 crystals were transferred to cryo solution containing reservoir, 25% (w/v) glycerol, peptide and piceatannol and frozen in liquid nitrogen. Sirt5 crystals were frozen after adding two drop volumes 15% (w/v) glycerol in reservoir solution supplemented with peptide and resveratrol.

Diffraction data sets were collected at BL14.1 operated by the Helmholtz-Zentrum Berlin (HZB) at the BESSY II electron storage ring (Berlin-Adlershof, Germany) with a MX-225 CCD detector (Rayonix, Evanston, USA; Sirt3) and in house on a Mar 345db (Marresearch, Norderstedt, Germany) image plate system (Sirt5). Indexing, scaling and merging of diffraction data was done with XDS [49]. Sirt5 crystals belonged to space group P2_1_ and showed pseudo-merohedral twinning (twin fraction 47%). Detection and refinement of twinning fractions was done with Sfcheck [50], Phenix [51], detwin [52], Refmac [53] and the Yeates twinning server at http://nihserver.mbi.ucla.edu/Twinning/ [54] (Figure S1). Structures were solved through Patterson searches with Molrep [55] and Phaser [56], with Sirt3 [43] and Sirt5 [42], respectively, as a search model. The model was refined with Phenix (Sirt5) or Refmac (Sirt3) with individual isotropic Debye-Waller factors, and rebuilding was done in Coot [57]. Parameter files for the FdL1 fluorophore, resveratrol, and piceatannol were generated with PRODRG [58]. For the FdL1 fluorophore, the 7-amino-4-methylcoumarin structure commonly used for this type of assay [59] was assumed. For validation of the structures, Coot and ProCheck [60] were used. Structural figures were generated with PyMol (http://www.pymol.org) and MGLTools [61].

### Co-Ordinates

Co-ordinates and structure factors of Sirt5 in complex with FdL1-peptide and resveratrol and of Sirt3 in complex with FdL1-peptide and piceatannol have been deposited with the Protein Data Bank (PDB accession codes 4HDA (Sirt5) and 4HD8 (Sirt3)).

## Supporting Information

Figure S1 Yeates plot for the Sirt5 diffraction data set. (PDF)

Figure S2 Electron density analysis of properly and randomly placed Sirt5 Ligands. (PDF)

Figure S3 Effects of tyrosol, quercetin, and fisetin on Sirt3 and Sirt5 activity. (PDF)

Table S1 Chemical structures of the compounds studied and their effects on recombinant human Sirt3 and Sirt5. (PDF)
