# Supplementary material for: A Molecular Mechanism for Direct Sirtuin Activation by Resveratrol
Source: PLoS One. 2012 Nov 21;7(11):e49761. doi: 10.1371/journal.pone.0049761 (PMC3504108; doi:10.1371/journal.pone.0049761)
Supplement: Figure S1 — (PDF) [file pone.0049761.s001.pdf]

Supplementary Figure S1

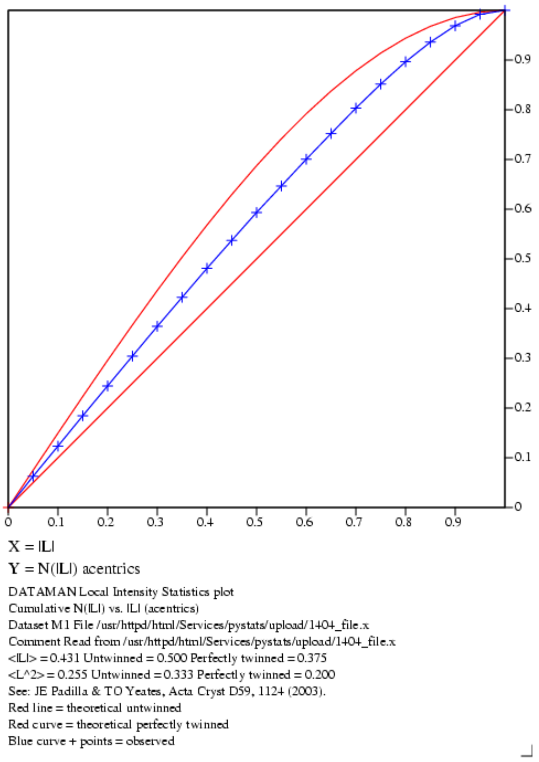

Supplementary Figure S1: Yeates plot for the Sirt5 diffraction data set. Yeates plot [1] for the diffraction data of the Sirt5/FdL-1/resveratrol complex, calculated with the Yeates twinning server [2], indicating twinning with a high twin fraction.

References

1. Padilla JE, Yeates TO (2003) A statistic for local intensity differences: robustness to anisotropy and pseudo-centering and utility for detecting twinning. Acta Crystallogr D Biol Crystallogr 59: 1124-1130.
2. Yeates TO (1997) Detecting and overcoming crystal twinning. Methods Enzymol 276: 344-358.
