# Supplementary material for: A Molecular Mechanism for Direct Sirtuin Activation by Resveratrol
Source: PLoS One. 2012 Nov 21;7(11):e49761. doi: 10.1371/journal.pone.0049761 (PMC3504108; doi:10.1371/journal.pone.0049761)
Supplement: Figure S2 — (PDF) [file pone.0049761.s002.pdf]

Supplementary Figure S2

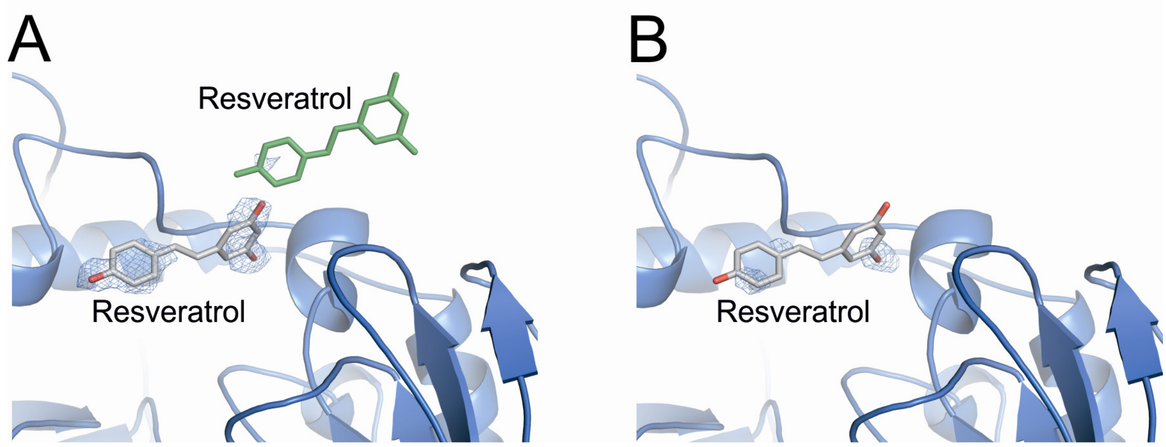

Supplementary Figure S2: Electron density analysis of properly and randomly placed Sirt5 ligands. A 2Fo-Fc density at 1.0σ (blue) defines the resveratrol pose (colored by atom type). For comparison, 2Fo-Fc density for a randomly placed resveratrol molecule (green) is shown at the same σ level. Both resveratrol positions were refined as individual complexes, and ligands and corresponding densities were merged in this figure. **B** 2Fo-Fc omit map (0.7σ) for resveratrol in the Sirt5/FdL-1/resveratrol complex. The density defines ligand positions only weakly, likely due to low occupancy, as saturating conditions could not be achieved. The structure could be equally well refined into a ligand complex and ligand free Sirt5, indicating a crude estimate of ~50 % for the occupancy for both, peptide and activator. The significance of the developing 2Fo-Fc density for the ligands is documented in panel A and confirms the placement of the ligands based on omit density.
