# Supplementary material for: A Molecular Mechanism for Direct Sirtuin Activation by Resveratrol
Source: PLoS One. 2012 Nov 21;7(11):e49761. doi: 10.1371/journal.pone.0049761 (PMC3504108; doi:10.1371/journal.pone.0049761)
Supplement: Figure S3 — (PDF) [file pone.0049761.s003.pdf]

Supplementary Figure S3

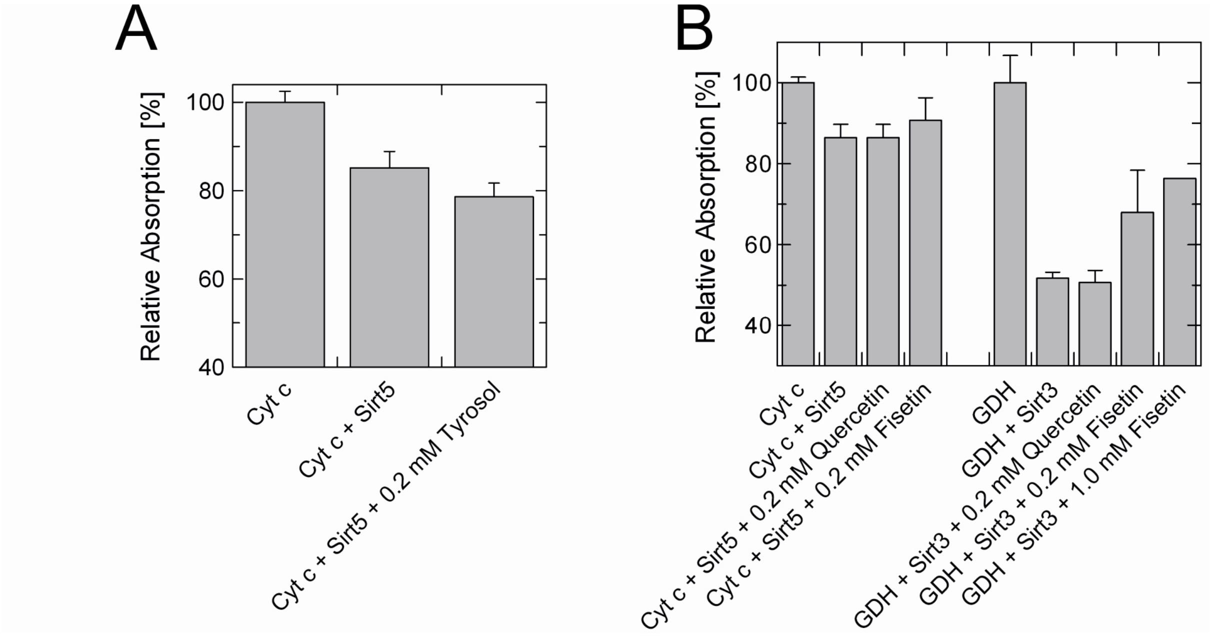

Supplementary Figure S3 Effects of tyrosol, quercetin, and fisetin on Sirt3 and Sirt5 activity. **A** Sirt5-dependent deacetylation of Cytochrome c (Cyt c), determined in an ELISA (deacetylation decreases absorbance), can be stimulated by tyrosol. **B** Quercetin does neither affect Sirt5-dependent decaetylation of Cyt c nor Sirt3-dependent deacetlyation of glutamate dehydrogenase (GDH) whereas fisetin has a weak inhibitory effect on Sirt5 and significantly inhibits Sirt3. Error bars represent standard deviations.
