# Supplementary material for: A Molecular Mechanism for Direct Sirtuin Activation by Resveratrol
Source: PLoS One. 2012 Nov 21;7(11):e49761. doi: 10.1371/journal.pone.0049761 (PMC3504108; doi:10.1371/journal.pone.0049761)
Supplement: Table S1 — (PDF) [file pone.0049761.s004.pdf]

Table S1: Chemical structures of the compounds studied and their effects on recombinant human Sirt3 and Sirt5

Compound	Chemical structure	Effect on Sirt3 activity		Effect on Sirt5 activity		
		FdL assay	ELISA	FdL assay	ELISA	Mass spectrometry
Resveratrol	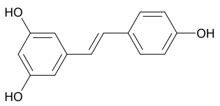	inhibition	inhibition	activation	activation	activation (Prx1 peptide and protein)<br>no effect (p53 peptide)
Piceatannol	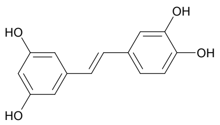	inhibition	n.d.	activation	activation	activation<br>(Prx1 peptide)
Tyrosol	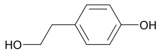	n.d.	n.d.	n.d.	weak activation	n.d.
Quercetin	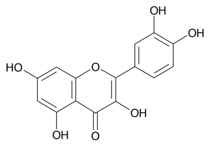	n.d.	no effect	n.d.	no effect .	n.d.
Fisetin	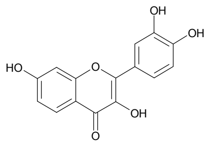	n.d.	inhibition	n.d.	weak inhibition	n.d.

n.d.: not determined
